# High prevalence of ECG variations and abnormalities in young and healthy TaMoVac 01 HIV vaccine trial volunteers from Tanzania

**DOI:** 10.1186/1742-4690-9-S2-P114

**Published:** 2012-09-13

**Authors:** PJ Mann, P Munseri, B Kaluwa, M Missanga, J Lwakatare, M Hoelscher, M Bakari, M Janabi, L Maboko, E Sandström, A Kroidl

**Affiliations:** 1 Mbeya Medical Research Programme, Mbeya, Tanzania, United Republic of; 2 Muhimbili University of Health and Allied Sciences (MUHAS), Dar es Salaam, Tanzania, United Republic of; 3 Department of Tropical Medicine and Infectious Diseases, University of, Munich, Germany; 4 Muhimbili National Hospital, Dar es Salaam, Tanzania, United Republic of; 5 Karolinska Institutet, Stockholm, Sweden

## Background

Vaccinia immunizations have caused peri-/myocarditis. As a result, volunteers receiving Modified Vaccinia Ankara(MVA) are monitored with ECGs. Early Repolarization Syndrome(ERS) has been reported in 1-2% and Left Ventricular Hypertrophy(LVH) in <10% of young, healthy populations across different ethnicities. These changes have been described as normal ECG variations, and according to early reviews, are more frequent in African and Asian populations. A prevalence of 90% has been reported for ST-Elevation in young males in several studies from industrialized and developing countries.

## Methods

ECG was performed in healthy HIV negative volunteers during screening for the Phase II ”TaMoVac01” HIV Vaccine Trial in DarEsSalaam and Mbeya, Tanzania. ECG variations that could potentially interfere with the later interpretation of myocarditis/pericarditis were confirmed by a panel of international cardiologists and led to exclusion from the study. These were ST-segment elevation, T-wave abnormalities, signs of LVH and ERS.

## Results

263 Volunteers (mean age 24.4 years, 63.5% males) had a baseline ECG evaluation performed. 19% of ECGs showed ERS, 20.3% showed LVH and 77% showed ST-elevation. 22.1% of volunteers were screened out due to ECG findings, none of whom had a history of cardiac disease, although one participant had a systolic murmur which led to echocardiogram. Dilation of the left ventricle was diagnosed.

## Conclusion

Although the prevalence of ST elevation was no higher than expected in young males, ERS and LVH was far more common than the literature suggested in this cohort of clinically healthy, young Tanzanians.Exclusions were due to the presence of more than one abnormality. None had symptoms that were clinically relevant, although a significant cardiac finding was revealed through echocardiogram in one participant.

The clinical benefit of ECG screening in the context of vaccine studies in healthy volunteers remains to be determined, but it is clear from this study that it added considerably to the number of screenouts.

